# Supplementary figures and images for: Opponent processes in visual memories: A model of attraction and repulsion in navigating insects’ mushroom bodies
Source: PLoS Comput Biol. 2020 Feb 5;16(2):e1007631. doi: 10.1371/journal.pcbi.1007631 (PMC7034919; doi:10.1371/journal.pcbi.1007631)

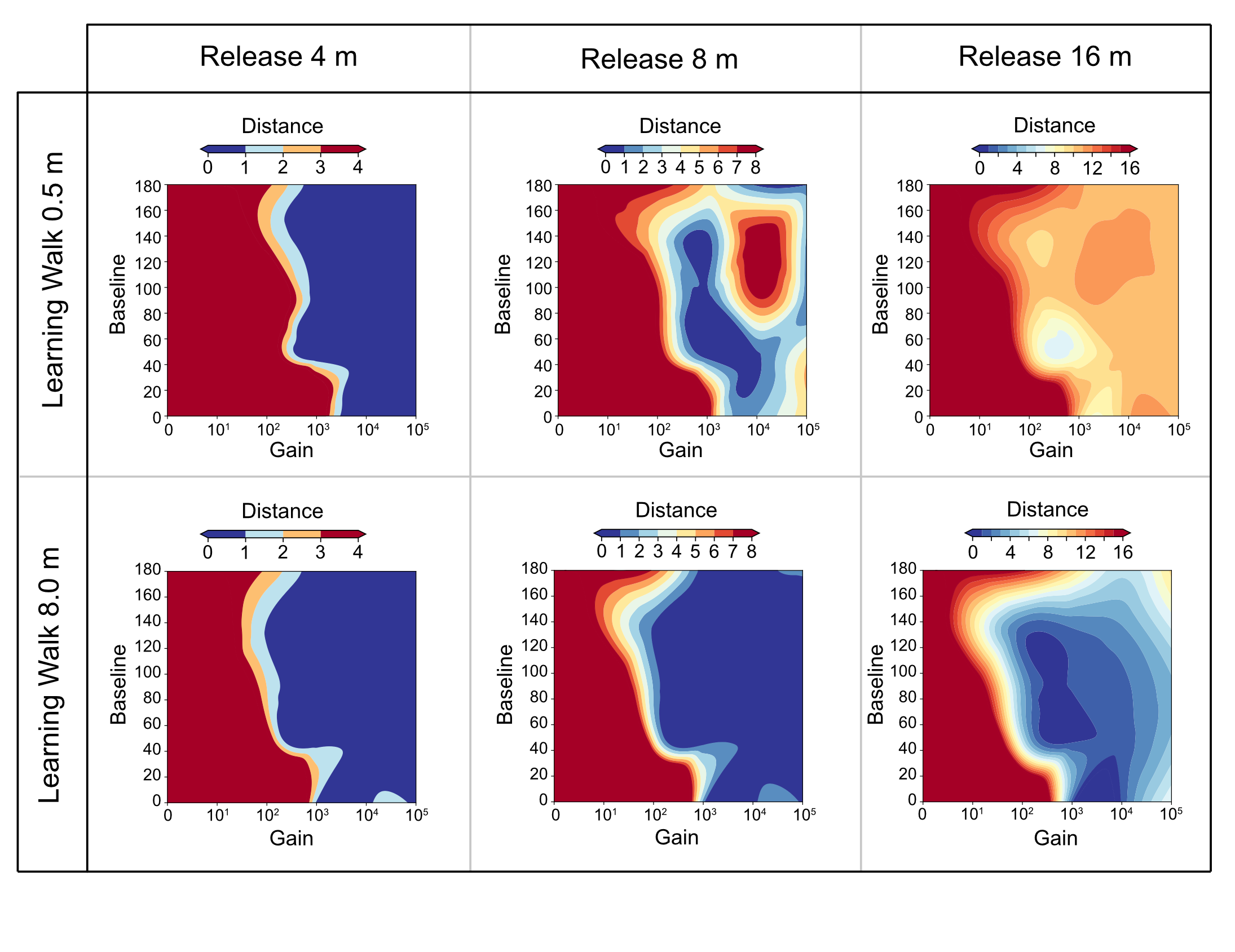

Supplement: S1 Fig — Heatmaps of the interaction between the gain and baseline parameters, and their effect on homing success (distance of arrival at the end of the simulation), for two sizes of learning walks and three release distances. (TIFF) [file pcbi.1007631.s001.tiff]

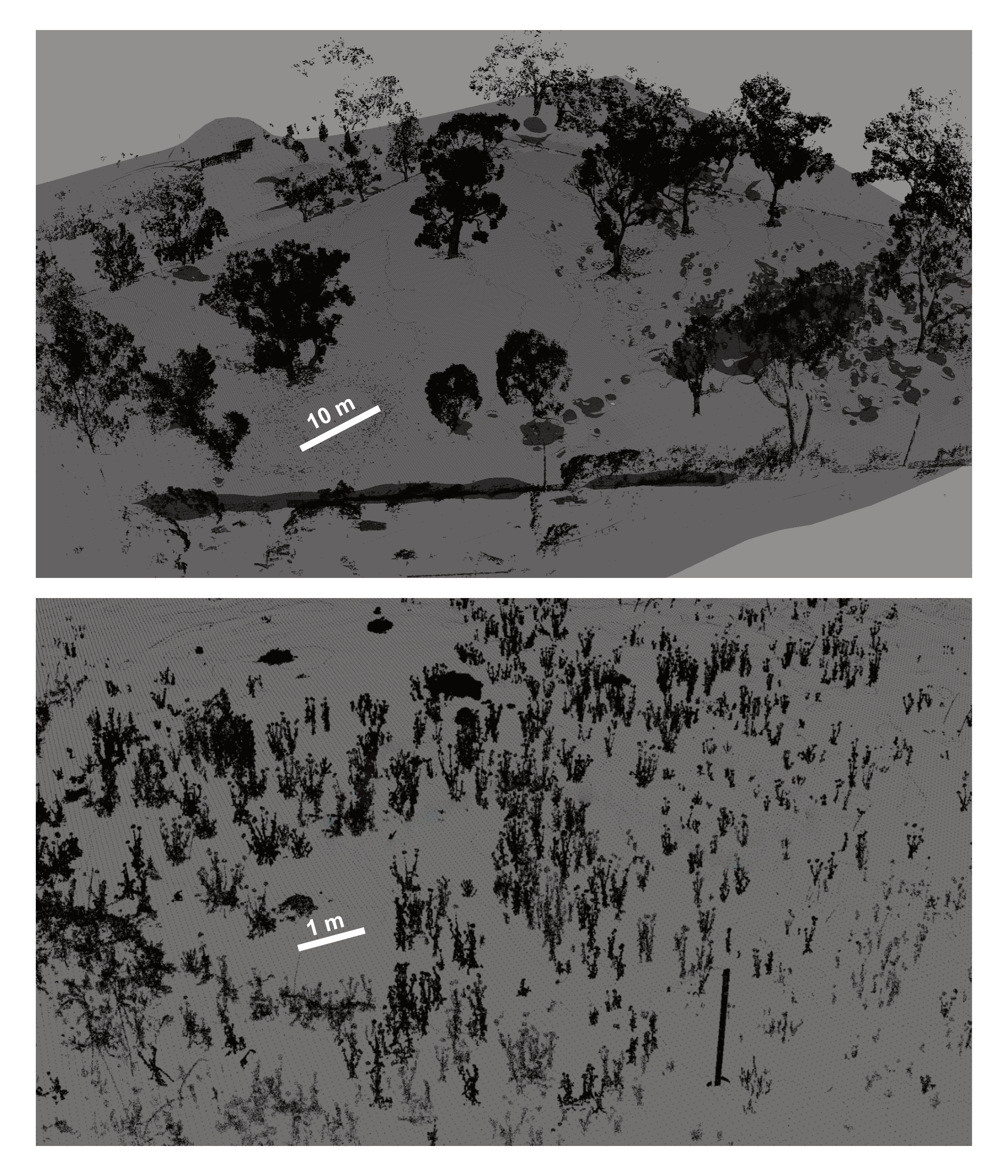

Supplement: S2 Fig — Snapshots of the 3D point clouds of the two virtual environments used in this work. Top, Canberra environment, large scale and large distant features such as trees; Bottom, Sevilla environment, with high clutter and no distal panorama. (TIFF) [file pcbi.1007631.s002.tiff]
