# Supplementary material for: Opponent processes in visual memories: A model of attraction and repulsion in navigating insects’ mushroom bodies
Source: PLoS Comput Biol. 2020 Feb 5;16(2):e1007631. doi: 10.1371/journal.pcbi.1007631 (PMC7034919; doi:10.1371/journal.pcbi.1007631)
Supplement: Attachment — Submitted filename: Response to reviewers 01.docx [file pcbi.1007631.s003.docx]

**We are grateful to the editor and the two reviewers for their time and comments. The manuscript has been significantly revised in the light of the two reviewers’ comments. We also re-ran some conditions to add a quantification of the models’ success in figure 4.**

**Below our response to the reviewers’ comments are in bold. All modifications brought to the manuscript are highlighted in yellow in both the manuscript and our answer to the reviewers.**

Reviewer's Responses to Questions

**Comments to the Authors:
Reviewer #1:**

This manuscript proposes the novel idea that central foraging insects learn and use ‘opponent’ visual memories to navigate. The core idea of ‘repulsive’ visual memories is intrinsically interesting.

Yet, my main concern is that it is partly motivated by inaccurate interpretation of former works and hasty claims. In particular, the fundamental drawback of the current models, as criticized by the authors, exists only if one assumes that insects can recognize a view only when properly aligned. This hypothesis might be supported by some data, but remains to be clearly demonstrated. Thus, “current models” indeed do not satisfy or even mention this precondition.

**Response: We agree that other ‘current models’ exist, some of which assume for instance the existence of ‘mental rotation’ (the author AW being himself co-author on such a paper, ref 57), and thus suffer from other types of drawbacks. The ‘current models’ that we refer to were not selected for their efficiency, but because they have been designed so as to be most biologically plausible in the sense that they can be implemented given the currently known neural circuitry (contrary to mental rotation models for instance, for which we do not see yet how this could be implemented neutrally).**

**We did not make an ‘inaccurate interpretation of former works’, but simply decided to focus on these types of models, which indeed assume that views are recognised only when properly aligned, and which do suffer from the fundamental drawbacks that we pointed out.**

**In the light of reviewer 1 comments (see our detailed response below), and to avoid further misunderstanding, we added a section in the introduction to enable the reader to get a quick overview of other models, as well as understand that we focused here only on the ones that fit with the known insect circuitry (line 79-94).**

Another claim is that the present study would propose a likely neural implementation. I am not convinced that the cartoon of a putative single inhibitory synapse (Fig. 1C) and mere parallel with Drosophila’s olfactory and appetitive neural system (l. 395-423) are sufficient to qualify as a likely neural implementation.

**Response: The implementation we suggest is not so ‘far-fetched’ given that it is the one actually described in insects, and not only in drosophila! The MB overall circuitry is remarkably conserved across insects (e.g., data from locusts, bees, ants, flies, larva), and it is known that massive visual projections to the MBs have evolved in hymenopterans, inlcuding ants, for instances:**

***Gronenberg. "Modality-specific segregation of input to ant mushroom bodies." Brain, behavior and evolution 54.2 (1999): 85-95.***

***Gronenberg. "Subdivisions of hymenopteran mushroom body calyces by their afferent supply." Journal of Comparative Neurology 435.4 (2001): 474-489.***

**Also, this includes the fact that MBONs encode different valences (positive and negative), which is usually achieved by having MBONs releasing GABA vs. MBONs releasing acetylcholine (that is, essentially, connections of opposite signs, as illustrated in our cartoon). In addition to the many papers published on drosophila MBONs (e.g., Aso et al., 2015 2016 elife), here are a few studies mentioning such aversive learning in honeybees:**

***Jarriault, David, et al. "Dopamine release in mushroom bodies of the honey bee (Apis mellifera L.) in response to aversive stimulation." Scientific reports 8.1 (2018): 16277.***

***Schleyer, Michael, et al. "Associative learning of stimuli paired and unpaired with reinforcement: Evaluating evidence from maggots, flies, bees and rats." Frontiers in psychology 9 (2018): 1494.***

***Strube-Bloss, Martin F., and Wolfgang Rössler. "Multimodal integration and stimulus categorization in putative mushroom body output neurons of the honeybee." Royal Society open science 5.2 (2018): 171785.***

**In light of the reviewer’s comment, we decided to add a paragraph including these references about the MB of hymenopterans “line 123-136”**

**Overall, we believe the claim that the present study proposes a likely neural implementation is valid, and wish to keep it like so.**

Likewise, as most predictions (l. 425-453) rely on this far-fetched neural implementation, they sound more like long-term future research axes than actual experimentally-testable predictions.

**Response: We now clarified the prediction sections (line 480-515). We believe our predictions are testable, 3 of them behaviourally. A strong and precise neural prediction (such as predicting the existence and response of a particular neurons) is also worth keeping, given that novel techniques now enable to perform electrophysiology on insect navigating in virtual environment on air suspended treadmill. Also, the other neural prediction can already be experimentally tested, in ants or other species. For instance, by pharmacologically inoculating a GABA-receptor antagonist (it is even possible to target a specific receptor subtype) in the output region of the MBON. This technique has already been performed in our lab on the Mushroom Bodies of bees:**

***Devaud, Jean-Marc, et al. "Neural substrate for higher-order learning in an insect: mushroom bodies are necessary for configural discriminations." Proceedings of the National Academy of Sciences 112.43 (2015): E5854-E5862.***

***Boitard, Constance, et al. "GABAergic feedback signalling into the calyces of the mushroom bodies enables olfactory reversal learning in honey bees." Frontiers in behavioural neuroscience 9 (2015): 198.***

**(Further backing up this model in ants, we already know that blocking MBONs activity specifically disrupt visually-based learnt navigation in solitary navigating ants, but this work is under review elsewhere and thus cannot be cited yet).**

Major comments:

The authors begin their argumentation by describing what they call “a widespread orthodoxy” (l. 73) which they define by the consecutive statements (l. 73-80):
(i) “insects memorise these scenes in an egocentric way”
(ii) “the recognition of a memorised scene … is dependent on the insect’s current head direction”
(iii) “current view will appear familiar only when the insect is aligned in roughly the same direction that it was at the time of learning. If the insect is located at a familiar location, but its body is oriented in any other direction, its current view would be different and the location would appear unfamiliar.”

**We agreed to remove the term ‘widespread orthodoxy’, and now our introduction situates these models into a broader literature, and present a dedicated subheading to ‘familiarity-based-model’ (line 91) and ‘limits of familirity-based-model’ (line 143).**

Although (i), (ii) and (iii) are articulated by the authors as equivalent assertions, they are not in my opinion. (i) seems too strong as some authors would claim that all navigational information is combined on a geocentric map, and others have proposed that snapshots would be memorized with the celestial compass direction to allow, during later recall, proper alignment and comparison with the current view.

**Response: Sure, but again models assuming geocentric map are typically less parsimonious and do not propose clear realistic implementations based on the known circuitry in insects. Also, regarding models assuming the association between snapshot and celestial compass (here again there no clear and plausible neural implementation yet) and they fail to explain why insects recognise views even if said views are rotated in a ‘wrong’ compass direction, as evidence for instance in :**

***Graham, Paul, and Ken Cheng. "Ants use the panoramic skyline as a visual cue during navigation." Current Biology 19.20 (2009): R935-R937.***

(ii) is what makes such image-based homing possible. Even if mental rotation was used (see below), the amount of rotation required would depend on the current insect heading, and hence would allow for proper correction of the current heading. (iii) is not valid in the case of mental rotation (see below). Also, it seems unlikely for a lost ant to ‘hit’ a familiar route under the correct angle, and thus searching would be particularly ineffective.

Then it is claimed that current models solve that issue by a stop-scan-go strategy, that is condemned as being “cumbersome”, “non-parsimonious” and (biologically) “unrealistic”. This might stem from misunderstanding the models based on computing rotational image difference functions (RIDFs). As in (50, 51, 56), multiple RIDFs are computed from rotating the current view against the multiple memorized snapshots, and the direction to steer is then given by a weighted circular mean of the directions at the RIDF minima. However, physical rotation is not the only way to compute RIDFs. As discussed in the attached manuscript under review, mental rotation is also possible and has been suggested already by several authors. In fact, RIDF-based models like (51) leave open how RIDFs are computed. In principle, to find maximum-match directions, RIDFs could be sampled by physical and/or mental rotations, covering fully or partially 360°, in a systematic or a random way. A typical RIDF profile shows a more or less narrow and deep minimum difference (i.e. max familiarity) when both images align to the same (goal) direction, and plateaus at higher values elsewhere. In the present model, the combined ‘opponent’ RIDF (Fig. 1B) also show a maximum difference (i.e. min familiarity) in the anti-goal direction. The ‘opponent’ RIDFs thus present steeper gradients than the flatter profiles of standard RIDFs, hence, helping any algorithm to find peaks. This, I think, is what the authors implied with “recovering the correct direction through scanning … [by] increase[ing] the difference between incorrect and correct directions” (l. 241-242), albeit in a less clear way to me.

In the present manuscript, a single point of ‘opponent’ RIDF is sampled at each time step using oscillatory and noisy physical rotations. Proof of principle is given that this strategy is effective when the agent turns with an amplitude modulated by the sampled value, but I am not convinced that this approach outclasses the other models with respect to the three criteria mentioned by the authors:

**Response: We did not claim nor imply that our model ‘outclasses’ mental rotation models in terms of performance. However, such mental rotation have no yet plausible implementation regarding insect neural circuitry, and it struggle to explain why the insects would go into the pain of turning their body to face specific direction during learning walks or scanning behaviours.**

Cumbersomeness:
Unlike physical 360°-scans on the spot, mental rotation is not cumbersome as it could be done “en route”.
Parsimony:
Although performing systematic mental rotation continually may appear computationally “non-parsimonious” compared to physical rotation, learning goal-directed and anti-goal views seems somewhat superfluous for animals with nearly 360° field of view. Why would they have to turn back to learn the already visible opposite view? Can’t they learn both views at once?

**We do agree that assuming the existence of mental rotation would equally solve some of the problems (as we showed for instance in ref 57). However, we do not see how this could be implemented neurally. In contrast, the modelling work presented here shows that the addition of a single neuron (which is known to exist!), is enough for the model to make a huge improvement in regards to the previous models that fit the insect circuitry.**

**Also, ants seem not to ‘learn both views at once’ since (as mentioned in the intro and discussion) during learning walks, they perform regular alternation between nest-facing and anti-nest-facing views, in all species looked so-far.**

Moreover, anti-goal views seem somewhat redundant with goal-directed views acquired while spiraling around a place. In fact, goal-directed views already cover “opponent” directions that, like in (51), can compete to steer the animal.

**Response: But having both goal and anti-goal directed views create a fundamental difference with having only goal facing views all around the nest! As shown in figure 2 and 3 (which is basically a replicate of models 51, 56 etc), using only goal-directed views require a scanning step for being effective, whereas the addition of anti-nest views solve this problem. This is the point indeed.**

Admittedly, the strategy proposed by the authors is parsimonious in that it computes only one RIDF value at a time, but the agent can then only determine how much to turn, not in which direction to turn. Hence, it does “waste” information contained in the snapshot memories, namely the proper direction to steer. That is, the proposed approach is parsimonious in computation but not in memory.

**Response: We agree indeed. We do believe that is likely that ants can use views to determine whether to turn left and right (we actually have a coming experimental manuscript in preparation on this topic, which actually also exploits the opponent system as presented here). As explained in the text, the point of the current paper is not about the sheer efficacity of this specific agent, but about the benefits of storing views of opposite valence.**

**Line 442 “It is important to note that we did not wish here to explore thoroughly how this particular agent responds to these different variables. We rather wanted to show that the principle of an opponent process in visual memories provides robust information which can be directly exploited for successful navigation.”**

Biological plausibility:
Stop-scan-go strategy is not that biologically “unrealistic” as it was reported for ants released at unfamiliar locations (28, 54).

**Response: Yes, to the fact that ants scan (sporadically) in unfamiliar locations (actually ref 66, which was published 4 years after being presented in conferences) is what inspired these first ‘stop-scan-go’ models. However, what makes these models ‘unrealistic’ is that ants very rarely scan in familiar locations.**

Mental rotation is not “unrealistic” either, as a recent neural ring model (10), using a clockwise and counterclockwise-shifted neural mapping, can compare whether a left or right rotation would produce a better match between the current compass input and the path-integration memory.

**Response: We are very well aware of this Central Complex model for Path Integration, having ourselves (both authors) published an extension of this model (ref 11). However, we do not see how this could represent a neural implementation for the mental rotation of views. What is rotated in this Path Integration model is only the egocentric representation of the current direction, encoded by a single neuron in the Central Complex (by having its projection performing an offset of one column along the ring-attractor). Performing a mental rotation of the whole currently perceived view, is to our knowledge an entirely different matter, as it would mean that views are not only retinal snapshots, but actual oriented topographical representations of the world, from where a direction may be extracted after comparing to a temporary reference direction …all of which would seemingly happen before the MBONs (so, in the insect visual neuropils) and not in the Central Complex, in order to keep the current egocentric direction representation untouched. This of course may still exist in insects using views for navigation (hymenopteran), but we are not aware of any behavioural or neurobiological evidence up to now.**

Furthermore, the authors generalize their opponent-memory strategy to route navigation. As no “look-back” during route learning has been reported, they argue (especially in their other manuscript under review) for the use of outbound memories as opponent views during inbound journeys. However, this does not fit well with trained individuals following distinct inbound and outbound paths, to and from the same feeder (32). Intuitively, if ants were simply inverting attracting/repelling roles among the same set of visual memories, one would expect that each individual would eventually travel on a unique bidirectional path (per feeder).

**Response: This is an interesting point indeed; however, the use of outbound memories as opponent views during inbound journeys (and vice and versa) would not necessarily and always lead to unique bidirectional paths. This is because the first inbound path is largely driven by path integration, and the absence of turn back and look prevent the influence of the negative views stored during the outbound trip. However, such negative views would help an ant facing in the wrong direction to turn back towards the correct direction, as we now clearly state in our prediction line 510: “This could be first tested behaviouraly, by looking whether a homing ants released on its outbout route tend to be repulsed from the outbound direcition.”.**

**In the light of reviewer 1 major comments, we decided to add a paragraph about other models, including mental rotation, and explaining why we chose to focus on these type of models, which have the advantage to fit the known connectivity of insects. We hope this will clarify our point and prevent concerns such as these.**

Modelling studies have proposed several types of algorithms to explain how insect memorise and use views for guidance. While most models assume that guidance is achieved by comparison of the currently perceived view to a memorised view (although this is still debated (20,21)), they vary greatly on the way this can be implemented (see (22) for a comprehensive classification). For instance, some homing models need to store only one view at the goal acting as an attractor (4,23) while others require multiple memorised views acting as direction setters pointing along a learnt route (18,24,25) or towards the goal (26–28). Also, some models compare directly the raw visual input (29–32) while others are based on some specific visual processing such as distorting the current view (26), performing Fourrier transformations (33,34), or extracting and recognising specific features from the environment (4).

**Familiarity-based models**. In the last decade, a particular type of models, which we call here ‘familiarity-based’ has gained attraction for its simple approach, that yet provides good explanations of a variety of observed ants’ navigational behaviours (18,24,27,28), and is consistent with the insect’s neural circuitry (9,29).

Minor comments:

l. 149-150: “we implemented” but ref. 44 is not authored by any author of the present manuscript

**Response: Unclear indeed. We reformulated: “As a proof of concept, we implemented this opponent memory principle in a simplistic ant agent model”**

l. 248-279: The present model computes a difference, not a ratio. A difference is expected to scale with its operands, thus these paragraphs are not that clear to me.
**Response: Indeed. The paragraph is now clarified: Instead of ‘but their ratio stays alike’ we mention: ‘****but the sign of their difference stays alike’.**

l. 451-452: Motivation-based selection of memory banks has also been proposed and tested in:
- Hoinville T, Wehner R, Cruse H. Learning and retrieval of memory elements in a navigation task. In: Biomimetic and Biohybrid Systems. Springer; 2012; pp. 120-131.
- Cruse H, Wehner R. (2011) No need for a cognitive map: decentralized memory for insect navigation. PLoS computational biology, 7(3), e1002009.

**Response: We added these two references. Thank you, and apologies for this omission.**

l. 512-513: The definition of familiarity as the inverse of RMS pixel difference seems dubious as it is likely to explode to overly large values or infinity when views would match closely. Also, it does not fit with the range [0:1] displayed on the figures.

**Response: Indeed. We clarified this statement “We normalise unfamiliarity values to fit the range [0:1], and then subtract the obtained value to 1 in order to get what we thus call “familiarity””**

l. 595-599: Although this algorithmic formalization is fine, it might confuse some readers with the ‘stop-scan-go’ strategy criticized earlier in the text. The critical point here is that there is no ‘stop’ step, and the ‘turn on the spot’ step is not a scanning procedure (e.g. sampling RIDFs), but simply an action. I think an explicit note would help to clarify that.

**Response: We added the following precision: It is useful to note that the turn performed in rule 2 represents the direction in which the forward step will be performed, and not a turn performed independently from the forward motion as in ‘stop and scan’ models.**

Fig. 1B: Although familiarity is function of direction, using xy-plots instead of polar plots could be more readable and help to relate to standard RIDF plots (albeit familiarity is inverse of difference). Also, polar plots tend to compress the middle and enlarge the sides. Thus, the two peaks of the ‘integration’ curve are equivalent, yet the peak at -1 is shrunk compared to the other at +1.

**Response: We agree that xy-plots may be more readable on a quantitative aspect, however the idea of the Figure 1 was to provide a global and qualitative overview of the model’s idea, hence our choice for polar plots and very basic [-1 ; 0 ; 1] ticks. We think the global shape (and direction!) of the curves is the important message, rather than their absolute intensities.**

Fig. 2A: The arrows are too little to be seen. I am not sure what “directionality” refers to and if it is connected to the “specificity” described in the text.

**Response:** **The arrows are apparent in the high-resolution version of the figure. We had raised the issue of the low resolution of the figures in the pdf built by PlosCompBiol upon submission, and were told that high resolution versions could be downloaded by the reviewers. We feel sorry that the reviewer did not get the high-resolution figures.**

Fig. 2B: Choosing a viewing direction perpendicularly to the learned route is peculiar, why not directed along the route? How is calculated the familiarity between the memory bank and the current view? In other words, how one value is drawn from multiple familiarity values? The horizontal panels below are confusing as they represent vertical transects (especially when considered the horizontal route).

**Response: We precisely avoided route-directed views in order to only look at the apparent familiarity from unfamiliar viewing directions (i.e. places from where homing would be permitted only thanks to the learning walk information). Thus, even if the perpendicular direction was rather arbitrary, we could have chosen any direction non-parallel to the route, which all result in qualitatively similar results on the** **maps.**

**We added these precisions to the figure legend: “Although this direction (North-facing) was picked rather arbitrarily, any other direction provided qualitatively similar results, with one exception: route-directed views. We deliberately excluded this direction, to show the apparent familiarity as seen from unfamiliar viewing directions”**

**The familiarity value is obtained by comparing the current view with all views in the memory bank and selecting the one with the smallest difference. We clarified this in the legend: “Familiarity values (colours) represent the match between the north facing view at this location and the memory bank (the view that gives the smallest difference)”**

**Finally, we shortened the vertical dashed lines in order to make the transect position more evident on the map.**

Fig. 2C: “Deviation” may be preferred to “angular distance”.

**Response: Agreed. We changed it.**

Fig. 3 & 4: Quality of plots is poor, please avoid low-resolution bitmaps.

**Response: High resolution .tiff files were downloadable from the links provided at the end of the pdf. This is a limitation imposed by PlosCompBiol and we feel sorry about it.**

Fig. 3A,B: Giving just single examples for ‘decoupled memories’ and ‘noisy learning angle’ scenarios is unconvincing to assess the robustness of the model to these critical parameters.

**Response: We changed the figure in order to quantify the success. However, the main point of the current paper is not to realize a parameter exploration but only to provide an insight on the benefits of the visual opponent process.**

**-----**

**We are very grateful to reviewer 1 for his time and detailed comments, which surely helped improving our manuscript. We hope that our answers and amendments to the manuscript answer his concerns.**

**Reviewer #2:**

This model makes a very valuable contribution to understanding insect navigation. In particular, it brings into focus the potential importance of aversive mechanisms, even for performing behaviours that intuitively appear to be attractive. I think this will have a profound effect on our understanding of navigation, regardless of how accurate any of the details of the model turn out to be in the future.

The model builds on a family of models developed over the last 10-15 years to implement visual route following using a holistic familiarity encoding. This type of model has the great appeal that is simple and is consistent with what is known about insect mushroom bodies, where such memories are likely to be encoded.

Previous models of this sort have simply encoded a set of views towards the nest as attractive. The novel idea here is also to encode a set of views away from the route as aversive. The authors show that by combining both sets of views, an agent can navigate far more reliably. They show a variety of ways in which the robustness of homing is significantly improved. The effects are impressive.

The idea is implemented with a previously published oscillatory model of navigation that works effectively by turning away more from less attractive views. In previous models, the level of attractiveness has been determined simply from a familiarity measure. The novel idea here is that attractiveness is the difference between familiarity in the nest-ward (stored as an ‘attractive memory bank’) and familiarity in the anti-nest-ward direction (stored as an ‘aversive memory bank’). The authors’ novel idea of using both an attractive and an aversive memory bank could potentially also be usefully adapted for other types of models.

Suggestions:

In the Introduction, it might be useful to make it clearer that there are many classes of models for visual homing in insects, and that your discussion of limitations apply to the class that your model comes out of.

**Response: Very good suggestion indeed. We have added such a paragraph in the introduction (line 79-94)**

In the Discussion, it might be helpful to note that with this new addition, this school of model can now do some of what a different class of ‘correspondence’ models (which unlike the present models require retinotopy to be preserved in the encodings) can do- namely home robustly from away from a route corridor.

**Response: We now mention in the intro:“For instance, some homing models need to store only one view at the goal acting as an attraor (4,23)”. Familiarity based model could already performing homing away from a route corridor, but required scanning, this is already mentionned at serveral other location in the manuscript.**

It is also interesting to note what this new model shares with the first computational model proposed by Cartwright and Collett. Although the two models are different in many key respects, they share the finding that guidance based on attraction alone is not robust, while one using a combination of attraction (to views indicating the nest is in front) and aversion (to views indicating the nest is behind) is robust.

**Response: We added the following citation in the conclusion: “Other models, such as ‘correspondence’ models (22), can also benefit from using both aversion and attraction components, this time across the visual field (4, 67). But these models have the limitation that retinotopy must be preserved in memory, which, contrary to familiarity-based model, does not fit the mushroom body connectivity.”**

As a general note about the writing style, try to avoid starting each paragraph by referring directly to the previous paragraph (e.g. ‘this problem’, ‘this hypothesis’, ‘in such a way’, ’Moreover’, ’However’). Certainly do not start new sections ‘in such a way’. It is much easier for readers if paragraphs, and particularly sections, are self contained.

**Response: This is very good advice; we used it for the new version of this manuscript and will keep this in mind for the future too!**

Line 112: “Limits of the current models.” It needs to be clear that you are referring to only a subset of the current models, as not all models have the limits your refer to. See also comment to line 129.

**Response: Indeed, we’ve now renamed them and used throughout ‘familiarity-based models’. And the subsection title ‘Limits of familiarity-based models’**

Line 119: insert “It is problematic [for these models] to infer … “

**Response: Done!**

Line 120: insert “In several [of these types] of models … “

**Response: The paragraph has been reworked: Typically, familiarity-based models eschew this problem by having the agent stopping and rotating on the spot to scan multiple directions at each steps;**

Line 129: “We present here a simple solution to this problem. … “ Referring back to the previous section, I think ‘this problem’ refers to the need to sample several directions in order to choose the best. In the implementation here, this problem is solved by using an oscillatory path and continuously modulating the amount of turning. From my reading, using the two memory banks solves a different problem that is addressed later: i.e. robustness. It might be helpful for the reader if you separated the two issues. Moreover, given that you are using an oscillatory model that is similar to previously published ones, it is perhaps not helpful to have the need for scanning in the ‘limits of the current models’, since (as I understand it - or possibly mis-understand it) you are not providing a new solution to that.

**Response: Indeed, we now refocused the rationale at solving the intrinsic problem of familiarity-based model: that familiarity does not correlates with directional error. Scanning and oscillations are both trying to eschew the problem, but do not solve it. “An alternative to scanning – based on lateral oscillations – has been proposed for route following but proves to be extremely sensitive to parameter change. This is expected as this alternative does not solve the intrinsic problem that current familiarity does not correlate with directional error.”**

Figure 2: There may well be arrows in this figure, but they’re not visible with the resolution of the pdf that I have. It would probably be worth making the arrows larger. Possibly that might require making the grid cells larger too.

**Response: The arrows are apparent in the high-resolution version of the figure. We had raised the issue of the low resolution of the figures in the pdf built by PlosCompBiol upon submission, and were told that high resolution versions could be downloaded by the reviewers. We feel sorry that the reviewer did not get the high-resolution figures.**

Line 214: This section should be called something like “The drawbacks of using only goal-oriented views [without scanning]”
You are imposing the constraint of facing in only one direction. So in line 216, change ‘while’ to ‘if’.
Line 221: insert “… [without scanning], familiarity does not reliable indicate … “
Line 229: As an example to my general point about writing style, start instead with something like “We show here that [the need for scanning] can vanish … “

**Response: Changes made.**

Figure 3: It is clear from this figure that the addition of the repulsive visual memories makes homing much more robust. But I find the details quite confusing, and in particular I don’t understand the pattern of failures for the attractive views only. What I understand, after spending a while trying to make sense of it, is that ants are trained at the grey dots, facing towards the nest, and along a nest-ward route indicated by the grey line (??). In this case, it makes some sense to me that the ants can home from along the route, and that they are not good at homing from the opposite side of the nest.

**Response: Yes indeed, the model was trained at the grey dots with the views facing towards the nest. We added this precision in the legend. The grey “line” is just an artefact of PlosCompBiol’s compression of the images, it is in fact composed of a series of grey dots (visible in the attached high resolution .tiff file).**

But it puzzles me why the trajectories on the opposite side of the nest appear to go nowhere at all. Is that because of the choice of the ‘optimal parameters’? Are optimal parameters chosen for each release site, and is optimality based on end distance from the nest? Thus choosing not moving about moving in the wrong direction?

**Response: Indeed. Optimal parameters were chosen after a parameter exploration at this given location in the virtual world, and success was based on the end distance from the nest of the end path. We added a sentence in the legend of figure 3: “Note that as a result of the poor success of this model, agents staying at the starting point are favoured over agents straying further away.”**

I also don’t understand the difference between A and B. Are they trained at the same location but tested at different locations? The positions of the release sites look the same so I don’t think that can be it. Or is the entire experiment, including both training and testing, shifted to a new location for B? This would explain where there is some homing in the right hand panel of B. But then I don’t understand why there would be no homing from the route (if the grey line is indeed the route) in the left hand panel of B.

**Response: the whole experiment (including both training and testing) was shifted to a new location. The lack of success in the left hand panel is just a consequence of the structure of the world, which, in this location, might be ambiguous to the model. We now clarified this in the figure 3 legend: B. Paths of agent trained and tested at a second nest location in the virutal world, but using the parameter optimised for the location in A.**

What is it about the parameters and different location that make so many trajectories head off towards the NE? In many of the panels in Figures 3&4 there is something special about the SW-NE direction. Why?

**Response: Given that all agents of a same plot have the same set of memory (except in the case where we add directional noise to the memories), unfortunate spurious matching can lead them to follow a same wrong direction.**

If the parameters are so critical to the success of using attractive views only, please try to describe what is needed more fully. Why some parameters work, why others don’t, and why the difference when shifting to a new location.

**Response: This explanation was already apparent in the previous manuscript, to which we added a sentence to link it to sensitivity to parameter change (line 313 to 320): “Absolute mismatch values resulting from the comparison of two views are dependent on the structure of the scenes. Locations presenting rather homogenous visual patterns across azimuths tend to yield low mismatches, while heterogonous scenes tend to yield high mismatches. For instance, if an agent ends up unusually close to a tree, the mismatch experienced may become unusually high, that is, the familiarity becomes unusually low (see for instance Figure 2B, left). Thus, what should be considered as a good or bad familiarity value is a tricky question, and this is why models using only attractive view without scanning are very sensitive to parameter calibration (Figure 3).”**

Also, why was there no problem in getting homing in references 28 and 41 referred to in the Introduction?

**Response: Because these agents in ref 28 and 41 where performing a stop and scan at each time step.**

Figure 4 shows nicely how robust the opponent-based navigation is.
C. insert: “half [as many] memories”
D. [Lower] resolution

**Response: Done**

Lines 444-448: This prediction appears to be counter to the findings of Schwarz et al 2017, in which the same authors found that using route memories to travel backwards required ‘peeking’ along the route to set the direction. Can you reconcile the previous findings with the current prediction?

**Response: It can be reconciled. This is explained in detail in our second ‘backward’ manuscript (still under review), which pdf was added to this submission. Long story short: in our 217 paper, the route was one way (a square). In our second paper under review, the route was a two ways (one same leg) back and forth. Only in this second case could the ant direct their path while going backward (without peeking).**

**–--**

**We are grateful for Reviewer2’s time and thourgough review.**

**Have all data underlying the figures and results presented in the manuscript been provided?**
Large-scale datasets should be made available via a public repository as described in the *PLOS Computational Biology* [data availability policy](http://journals.plos.org/ploscompbiol/s/data-availability), and numerical data that underlies graphs or summary statistics should be provided in spreadsheet form as supporting information.

Reviewer #1: No:

Reviewer #2: Yes

PLOS authors have the option to publish the peer review history of their article (what does this mean?). If published, this will include your full peer review and any attached files.


If you choose “no”, your identity will remain anonymous but your review may still be made public.


**Do you want your identity to be public for this peer review?** For information about this choice, including consent withdrawal, please see our [Privacy Policy](https://www.plos.org/privacy-policy).

Reviewer #1: No

Reviewer #2: No

*In compliance with data protection regulations, you may request that we remove your personal registration details at any time.* [*(Remove my information/details)*](https://www.editorialmanager.com/pcompbiol/login.asp?a=r)*. Please contact the publication office if you have any questions.*
