# Supplementary material for: Opponent processes in visual memories: A model of attraction and repulsion in navigating insects’ mushroom bodies
Source: PLoS Comput Biol. 2020 Feb 5;16(2):e1007631. doi: 10.1371/journal.pcbi.1007631 (PMC7034919; doi:10.1371/journal.pcbi.1007631)
Supplement: Attachment — Submitted filename: Response to reviewers_round2.docx [file pcbi.1007631.s004.docx]

Reviewer's Responses to Questions

**We first would like to thank the reviewers for their comments and the time taken to read this second version of our manuscript. Our response is below in bold. Changes made are highlighted in yellow in our reviewed manuscript, uploaded as an attachment.**

**Comments to the Authors:**


Reviewer #1: Thanks for your edits. Focusing explicitly on a specific class of models now sounds fairer. However, “familiarity-based models” seems inaccurate. “Image rotation” or more precisely “Rotational Image Difference Functions” (RIDFs) is a central concept (although never mentioned in the paper) to several familiarity-based models. In several cases, e.g. ref. 28, the model is agnostic as to how RIDFs are computed. It could be done by on-the-spot scans as criticized in the paper, or it could be done by some mental rotation (also not mentioned in the paper) as I already objected. It would be more accurate to call the addressed class of models “stop-scan-go models” and describe them as a certain way, namely embodied image rotation, to implement familiarity/RIDFs-based models.

**A: It is true that many familiarity-based models use RIDFs, full or partial, but rotations are not crucial to familiarity based model (see 44). The solution presented in this paper spares the need of computing rIDF (physically or mentally), but still shares the key features of ‘familiarity based models’ that we mention. Thus we believe the general class of models we are concerned here remains ‘familiarity based model’ rather than ‘stop-scan-go models’. We now mention that most familiarity based models use a ‘stop-scan-go’ strategy.**

Reviewer #1: Perhaps, the authors do not see how mental rotation could be neurally implemented, and it seems indeed less parsimonious (neurally) than a simple image difference, but to my knowledge there is yet no evidence excluding mental image rotations.

**A: it is indeed difficult to see how mental rotation could be implemented in the insect brain. Such a feat would at least require that visual encoding is achieved on the 360 visual field at once (as do models of rotation (34)), but ants, bees and wasp visual neuropils in each hemisphere seem to receive almost purely projection from the ipsilateral eyes only, which visual field cover around 180 degrees only. We added a paragraph in the introduction to explicit the reasons why we did not want to assume mental rotation.**

Reviewer #1:To defend that they would propose a “likely neural implementation”, the authors rely on the following reference-supported arguments: mushroom bodies (MBs) are well conserved across species; MBs process olfactory and visual inputs; MBs project excitatory and inhibitory outputs; and MBs are crucial for appetitive and navigation behaviors. But why, within MBs, the olfactory circuits would indicate anything about the visual circuits, furthermore when considered in two species subject to quite different contexts, walking vs. flying, foraging centrally vs. non centrally, social vs. non social? Inhibitory and excitatory neurons are common to quite many brain areas, including MBs. Using that as supporting that MBs store views in an opponent way appears far-fetched to me.

**A: The MB circuitry for visual neurons is not based on the description about olfactory neurons, but on the actual description of MB visual neurons (some KCs are purely olfactory, other purely visual, and in some ants, a few KCs are both). Also the output of the MB is not only inhibitory or excitatory, but convey valence information and can actually trigger appetitive (going straight) and aversive response (turning). These points are stated clearly in the end of our introduction:** *“in insects’ brains, it is now clear that several output neurons from the Mushroom Bodies (so-called MBONs), which typically respond to learnt stimuli, convey either attraction or avoidance signals (58,62–64); and that these MBON signals with opposite valences are integrated downstream (65–67).”*

**Finally, MB size variation across species appears to correlate with the need for navigation (rather than walking vs flying or social vs. non social), this is discussed in the paper we cite (9), but we feel this point is not relevant enough for the current study to be explicated in the manuscript.**

Despite those considerations, I recommend this paper for publication.

Reviewer #2: This manuscript is now much improved and I have only a few minor comments. As before, I think that the model has the potential to have a big impact on how we think about navigation in insects, and so is well worth publishing here.

line 29: compares

**A : Done**

line 126-7: “retinotopy does not need to be preserved for the comparison stage”. I’m not sure this is necessarily correct. I think you mean that you don’t need a retinotopic mapping.
**A : replaced b**y **‘Familiarity-based models do not need retinotopic mapping to be preserved at the comparison stage’**

line 145: “some drawbacks”. this paragraph seems to be about one drawback.
**A : replaced by ‘one main drawback’**

line 169:”the problem of familiarity-based models”. You seem to be saying here that familiarity-based models are a problem in themselves - which is obviously not what you mean.

**A : Indeed, we now mention ‘previous familiarity based models.**

Line 173-4. Since you make such a big thing of it in the next sentence, this particular assumption that both outputs are continuously integrated does appear to be made through the desire to improve a model rather than flowing from observations.

**A : The assumption about downstream integration does result from neurobiological observations, as mentioned later on: “these MBON signals with opposite valences are integrated downstream (65–67)”. We nonetheless removed ‘continuously’ from our assumption, this results from the desire to make the simplest model.**

line 184: “this hypothesis” is that the solution you present in the previous paragraph is correct?
**A : replaced by ‘the effect of these assumptions’**

Lines 220-270 appear to be more or less repeating lines 90-167. Can they be omitted or merged into the existing Introduction?
**A : We agree that this present some redundancy with the introduction, but now while bringing in our analysis and calling for results figures. So this ‘result part’ could not be moved up into the introduction. One alternative could be to shorten the introduction, but given the importance of understanding the concept presented here, we preferred to keep it as it is.**

line 277: subtracted from
**A : Done**

line 281: omit ‘rather’
**A : Done**

line 356: ‘the’ instead of ‘this’
**A : Done**
